# Asthme professionnel indemnisé dans le centre tunisien: étude ransversale sur huit ans

**DOI:** 10.11604/pamj.2017.26.164.11486

**Published:** 2017-03-21

**Authors:** Amira Omrane, Awatef Kreim, Mohamed Adnène Henchi, Selma Kammoun, Leila Bessadi, Charfeddine Amri, Taoufik Khalfallh, Lamia Bouzgarrou

**Affiliations:** 1 Département de Médecine de Travail et d’Ergonomie, Faculté de Médecine de Monastir, Monastir, Tunisie; 2 Caisse nationale d’Assurance-maladie de Tunisie, Tunisie

**Keywords:** Asthme professionnel, déclaration, incapacité, Occupational asthma, declaration, disability

## Abstract

**Introduction:**

Les objectifs étaient de dresser le profil épidémiologique des patients indemnisés pour asthme professionnel dans le centre tunisien, identifier leurs caractéristiques professionnelles et évaluer les pratiques d’indemnisation de cette maladie professionnelle.

**Méthodes:**

Étude exhaustive rétrospective, menée sur une période de huit ans, à propos des cas d'asthme professionnel reconnus et indemnisés au centre tunisien par les deux seules commissions médicales habilitées à fixer le taux d’incapacité partielle permanente aux victimes des accidents de travail et des maladies professionnelles dans les sept gouvernorats du centre.

**Résultats:**

Au total, 129 travailleurs, d’âge moyen égal à 40,6 ± 7,75 ans étaient indemnisés pour asthme professionnel durant la période d’étude. Le sexe ratio était de 0,66. Les agents étiologiques les plus incriminés ont été les poussières végétales textiles (75,2%), les poussières de bois, la farine et les isocyanates. Une hyperréactivité bronchique non spécifique a été retrouvée dans 38%, un taux élevé d’IgE dans 14% et des prick tests positifs dans 10,9% des cas. Le taux moyen d’incapacité partielle permanente était de 25,6±14,2%. Ce taux était corrélé, selon l’analyse analytique, à l’âge du patient et à la commission médicale ayant fixé ce taux.

**Conclusion:**

L’étude des cas d’asthme professionnels indemnisés, apportent des données pertinentes à propos des caractéristiques épidémiologiques et cliniques des patients atteints et des pratiques des comités notamment, en terme d’application du barème indicatif, mais ne permet pas l'appréciation de la prévalence, souvent sous-estimée de cette pathologie.

## Abstract (en)

**Introduction:**

Our study aimed to highlight the epidemiological profile of patients compensated for occupational asthma in the Tunisian Center, to identify their professional characteristics and to determine compensation practices for this occupational disease.

**Methods:**

We conducted an exhaustive retrospective study over a period of eight years. This case study included workers with occupational asthma diagnosed and compensated in the Tunisian Center by the only two medical commissions empowered to set rates for permanent partial disability caused by either a work-related injury or an occupational diseases in the seven central governorates.

**Results:**

A total of 129 workers, with average age of 40.6 ± 7.75 years were compensated for occupational asthma during the study period. Sex ratio was 0.66. The most incriminated etiologic agents were vegetable dust pollution in the textile industry (75.2%), wood dust, flour and isocyanates. Nonspecific bronchial hyperreactivity was found in 38% of cases, a high rate of IgE in 14% of cases and positive skin prick test in 10.9% of cases. The average rate of permanent partial disability was 25.6 ± 14.2%. On the basis of the analytical study, this rate was related to patient’s age and to the medical commission which had set this rate.

**Conclusion:**

This study of workers compensated for occupational asthma provides relevant data about epidemiological and clinical features of diagnosed patients and committee practices in particular, in term of application of the voluntary indicative scale, but it does not allow an assessment of the prevalence of this pathology which is often underestimated.

## Introduction

L’asthme professionnel (AP) compte parmi les pathologies respiratoires les plus fréquentes en milieu de travail [1]. Environ 10-25% des asthmes des adultes peuvent être attribués à l’asthme professionnel [2]. Si différents mécanismes physiopathologiques peuvent être incriminés dans le développement de cette pathologie, l’asthme allergique demeure le mieux étudié [2, 3]. Pourtant, quel que soit le mécanisme de l’AP, le diagnostic doit reposer sur une démarche structurée [4]. En Tunisie, comme dans plusieurs pays, une fois établi, ce diagnostic ouvre droit à une reconnaissance en maladie professionnelles selon les textes de réparation des maladies professionnelles en vigueur. Les objectifs de ce travail ont été d’identifier les caractéristiques professionnelles, de dresser le profil épidémiologique et clinique des patients indemnisés pour asthme professionnel dans la région du centre tunisien et d’évaluer les pratiques d’indemnisation de cette maladie professionnelle en Tunisie.

## Méthodes

Etude rétrospective exhaustive portant sur les cas d'AP reconnus et indemnisés au centre tunisien par les deux commissions médicales des accidents de travail et des maladies professionnelles de Sousse et de Monastir (Tunisie), habilitées à fixer les taux d’incapacité partielle permanente (IPP), au cours d'une période de huit ans, allant du 01 Janvier 2004 au 31 Décembre 2011. Une liste exhaustive des patients reconnus et indemnisés pour AP durant la période d’étude a été établie à l’aide de la base de données ORACLE de la Caisse Nationale d’Assurance Maladie (CNAM). Le recueil des données s’est basé sur une fiche préétablie de 36 items, complétée à partir des dossiers médicaux de déclaration de maladie professionnelle déposés auprès des bureaux régionaux de la CNAM et des rapports des enquêtes techniques réalisées par les ingénieurs de la CNAM faites à l’occasion de chaque déclaration d’asthme. Les données recueillies étaient relatives aux caractéristiques socioprofessionnelles et habitudes de vie des patients, ainsi que les antécédents généraux, respiratoires et d’atopie, personnels et familiaux; le tableau clinique de la pathologie; les résultats des examens complémentaires pratiqués, de même que les caractéristiques des expositions professionnelles et les données relatives à la procédure de la déclaration et l’indemnisation. Les explorations fonctionnelles respiratoires ont été interprétées en fonction des limites inferieurs de la normalité et des valeurs de références spirométriques établies et validées auprès de la population tunisienne [5]. Le syndrome obstructif a été défini selon l’American Thoracic Society et l’European Respiratory Society, comme un rapport entre le volume expiratoire forcé seconde (VEMS) et la capacité vitale lente (CV) inférieur à la limite inférieure de la normale (LIN) [5, 6].

## Résultats

### Etude descriptive

**Caractéristiques épidémiologiques et professionnelles:** Durant la période d’étude, 129 patients ont été indemnisés pour AP au centre tunisien, avec une incidence annuelle moyenne égale à 16,1 ± 12,5 cas (4 et 35 cas). Une prédominance féminine (61,2%) et de la tranche d’âge de 35-45 ans (47,3%) a été notée parmi ces patients. La consommation tabagique n’a été précisée que dans un cas sur deux (55,8%); 12 patients ont été des fumeurs. Concernant les caractéristiques professionnelles, la catégorie des ouvriers (qualifiés ou non) a regroupé 97,8% de la population étudiée. Les postes occupés, variables selon les secteurs d’activité, ont été prédominés par celui de couture sur machine (28,5%), d’ouvrier polyvalent (15,3%) et celui de contrôle qualité (13%). L’ancienneté moyenne au poste a été de 16,4 ± 9,3 ans. Une ancienneté comprise entre 5 et 15 ans a été notée dans 43,4 % des cas (Tableau 1).

**Tableau 1:** Caractéristiques socioprofessionnelles

Caractéristiques	Moyenne	Minimum	Maximum
Âge	40,8 ± 7,6 ans	22 ans	59 ans
Genre	79 femmes (61,2%), Sexe Ratio = 0,63		
Secteur d’activité	Confection - Textile (74,3%), Plastique (5,4%), Automobile (5,4%)		
Ancienneté professionnelle au poste	16,4 ± 9,3 ans	1	40

**Caractéristiques cliniques de l’asthme professionnel réparé:** Concernant les antécédent et les habitudes de vie, cinq patients indemnisés pour AP (3,8%) ont eu des antécédents familiaux d’asthme et 38 (soit 29,4%) ont eu des antécédents personnels d’atopie. Parmi ces derniers, la rhinite allergique a été la manifestation clinique d’atopie la plus fréquente (13,2% des 38 patients atopiques), suivie par l’eczéma de contact (5,4% des 38 cas), la dermatite atopique et la conjonctivite. De plus, dix patients présentés des antécédents de pathologies respiratoires, à type de broncho-pneumopathies chroniques obstructives (7 cas), tuberculose pulmonaire (2 cas), et de dilatation de bronche (1 cas). Le tableau clinique, a comporté des symptomatologies respiratoires isolée dans 38% des cas, et associé à une rhinite dans 37,2% des cas et à une conjonctivite dans 2,3%. L’AP a été découvert à l’occasion de l’exploration d’une rhinite allergique sans signes respiratoires d’asthme associés dans 22,5% des cas. Dans notre étude, 38% des patients ont présenté une symptomatologie respiratoire isolée à type de sifflement, de dyspnée, de gêne respiratoire, de toux irritative, d’oppression thoracique. Selon les données du certificat médical initial de la déclaration d’AP, les râles sibilants ont été notés lors de l’examen clinique chez 41 patients (31,8%). Un rythme professionnel défini par l’existence d’un lien chronologique entre les symptômes d’asthme et l’exposition professionnelle avec une amélioration pendant les périodes de repos hebdomadaires et/ou les congés annuels et une aggravation lors de la reprise de l’activité, a été confirmée dans 55,8 % des cas. Une dyspnée permanente en absence d’un rythme professionnel a été retrouvée chez neuf patients.

**Données des explorations complémentaires:** Les explorations respiratoires fonctionnelles de base (EFR), interprétés en fonction des valeurs de référence [**5**], ont été normales dans 38% des cas et ont objectivé un syndrome obstructif dans 60,4% des cas et un syndrome mixte dans 1,6% des cas. La réversibilité a été confirmée dans 94,8% des cas des syndromes obstructifs. Pour l’ensemble des patients, le VEMS moyen a été de 71,5±18,4% de la théorique (30-108), le DEM 75% était de 64,2±25,1% de la théorique; le DEM 50% était de 59,9±25,1% de la théorique et le DEM 25% de 58,1±22,61% de la théorique. Le test de provocation d’hypersensibilité non spécifique a été prescrit systématiquement chaque fois que les EFR de base étaient normaux. Les résultats ont été positifs dans 49 dossiers, avec une PD 20 moyenne de 421±370,5 μg de méthacoline. La mesure répétée du débit expiratoire de pointe (débitmètre longitudinale) n’a été pratiquée que dans quatre cas avec une durée minimale de mesure égale à trois semaines et un nombre minimum des mesures journalières égale à quatre. Dans l’ensemble, ces débitmètres ont objectivé une variation intra et inter journalières [7]. Le bilan immunologique demandé a été limité aux prick tests (27 cas) et au IgE totale (18 cas). Parmi les 129 dossiers étudiés, aucune prescription de dosage des IgE spécifique n’été notée (Tableau 2). Par ailleurs, une radiologie de thorax a été pratiquée chez 72 patients (55,8%), objectivant une distension thoracique dans 31 cas (24%). Une rhino manométrie a été réalisée chez 114 sujets objectivant une obstruction nasale chez 108 patients (83,7%), avec une réduction bilatérale des volumes dans 52,7% des cas.

**Tableau 2:** Répartition des travailleurs indemnisés pour asthme professionnel selon les résultats des examens complémentaires

Explorations	Effectifs	%
**Spirométrie de base (n=129, soit 100%)**		
Syndrome obstructif réversible	73	56,6
Syndrome obstructif non réversible	5	3,8
Syndrome mixte	2	1,6
EFR normale	49	38
VEMS= 71,5±18,4% [30 -108%]		
CVF= 76,6±17,3% [38 - 110%]		
Rapport Tifneau= 79,3±13,8 [48 – 125]		
DEMM= 59,1±23% [18 – 114%]		
DEM25%= 59,9±25,1% [19 – 114%]		
DEM50%= 58,1±22,6% [13 – 97%]		
DEM75%= 64,2±25,1% [16 – 113%]		
**Test de provocation non spécifique à la métacholine**		
Positive	49	38
PD_20_= 421±370 μg [50 – 1600 *μg]		
**Débimétrie longitudinale**		
Examen pathologique	4	3,1

**Déclaration et réparation:** Parmi les patients reconnus porteur d’AP, six, soit 4,7% des sujets, ont bénéficié d’une reconnaissance et indemnisation antérieure pour une pathologie professionnelle autre que l’asthme. La maladie en question a été une surdité professionnelle dans trois cas (avec un taux d’IPP accordé variable entre 35 et 45%), une rhinite professionnelle dans deux cas (taux d’IPP égal à 7 et 12% avec à la déclaration des agents étiologiques autres que ceux en cause de l’AP chez ces patients) et dans un cas un eczéma allergique au chrome (avec un taux d’IPP accordé égal à 10%). Concernant la déclaration et la reconnaissance de maladie asthmatique accordées durant la période d’étude, elle a été associée à la déclaration et la reconnaissance d’une rhinite allergique dans 45% des cas et à une conjonctivite d’origine professionnelle dans 2,3% d’entre eux. Parmi ces déclarations 65% ont été réalisées par un médecin du travail. Le tableau 53 des maladies professionnelles intitulé « les poussières textiles végétales » a été le tableau de déclaration dans 72,8% des cas. Le délai moyen écoulé entre la première consolidation et la date de convocation pour la commission de réparation habilitée à fixer le taux d’IPP était de 316 ± 221,7j. Le taux d’IPP moyen a été de 26,3 ± 13,8% avec des extrêmes variables entre 10 et 70% (Tableau 3).

**Tableau 3:** Caractéristiques de déclaration de maladies professionnelles

Caractéristiques	Moyenne
Médecin déclarant	Médecin de travail universitaire (42,6%); MT de l’entreprise (22,5%); Pneumologue (10,8%); Médecin généraliste (8,5%).
Agent étiologique	Poussières textiles (72,8%); Farine (5,4%) ; Bois (4,6%); Isocyanate (4,6%); Caoutchouc (3,1%), Acrylate (3,1%).
Délai consolidation-commission de réparation	316 ± 221,7 j; min = 146 j et max = 589 j
Taux d’IPP	26,3 ± 13,8%;min = 10%; max = 70%

**Étude analytique:** L’âge moyen des travailleurs de sexe féminin a été de 39,01 ± 6,77 ans alors que celui des travailleurs de sexe masculin a été de 47,78 ± 8,16 ans avec une différence statistiquement significative (p= 0,00). La différence de la répartition selon les tranches d’âge des travailleurs de sexe féminin et masculin a été aussi statistiquement significative (p = 0,002). Une étude statistique comparative a été conduite afin de comparer les pratiques entre les deux commissions régionales habilitée à fixer le taux d’IPP à Sousse et à Monastir. Alors qu’aucune différence statistiquement significative n’a été notée en terme de délais écoulés entre les dates de première consolidation et celle de la commission médicale (p= 0,8), une différence statistiquement significative a été notée en terme des taux d’IPP accordés par ces deux commissions (p= 0.003). Pour tenter de mieux cerner cette relation statistique entre les taux d'IPP accordés et la commission médicale ayant traité le dossier, et après vérification de la distribution normalienne des deux sous populations; nous avons procédé à une comparaison des moyennes et des variances des deux groupes de patients indemnisés par la commission de Sousse et de Monastir en se basant sur les tests test t de Student et F de Fisher, ainsi que les tests Chi 2 pour les variables qualitatives. Ces tests ont conclus que ces deux sous population sont comparables en termes de répartition selon le genre, 1es antécédents médicaux notamment l’atopie, les secteurs d’activité, l´ancienneté professionnelles et en termes de caractéristiques cliniques et d’explorations complémentaires des séquelles réparées. Finalement, nous avons procédé à une analyse multi variée, par régression multiple descendantes pas par pas afin de déterminer les corrélations entre le taux d’IPP et les variables d´intérêt. Le modèle statistique explicatif final a retenu que ce taux d'IPP n’était corrélé qu’à l'âge (p= 0.00) et à la commission médicale régionale qui a accordé ce taux (p= 0.024). L’ensemble des autres variables d’intérêt initialement introduite dans le modèle (genre, ancienneté professionnelle, l’association ou non d’une rhinite et ou d’une conjonctivite) et les données cliniques et d’explorations respiratoires de la maladie asthmatique indemnisée (VEMS, taux de réversibilité, PD 20) ont été exclues de ce model explicatif.

## Discussion

Cette étude a été confrontée, comme la majorité des études rétrospectives, à la non disponibilité de certaines données, particulièrement ceux liées aux résultats des explorations complémentaires. Le nombre moyen des cas d’AP réparés au centre tunisien étant de 16,12 cas/an, ne peut refléter qu’une estimation plutôt vague de la prévalence de cette pathologie dans la région. En effet, les principales sources d’information sont les statistiques de la CNAM, qui fournissent une idée sur les maladies professionnelles déclarées dans le secteur privé. Une étude national rétrospective des cas d’AP exerçant dans le secteur privé de 2000 à 2008 a retrouvé une incidence annuelle de 24,42 cas /1 000 000 travailleurs [8]. L’AP représente 7,17% de toutes les maladies professionnelles et 44,19% des maladies respiratoires professionnelles [8]. En Tunisie, dans le secteur public, une étude étalée sur quatre ans (2008-2011) a trouvé 76 cas de maladies professionnelles reconnus dont seulement trois cas d’AP (soit 3,9% du total des maladies professionnelles) [9]. La prévalence de l’AP dans le monde est variable. Une étude, impliquant près de 7000 participants dans 13 pays utilisant des méthodes uniformes pour identifier l´asthme d´apparition tardive, a montré que le risque attribuable à l´asthme professionnel était entre 10 et 25%, équivalent à une fréquence de 250 à 300 cas pour 1 million de personnes/ risques année [10]. Une analyse systématique du risque attribuable a montré qu´environ 16,3% de tous les cas d´asthme apparu à l’âge adulte sont causés par des expositions professionnelles [11].

### Profil épidémiologique et clinique

Dans notre étude, l´âge moyen a été 40,8 ± 7,6 ans avec une population d’étude plus tôt jeune dont 72% des sujets étaient âgés de moins de 45 ans. Selon les données de la littérature, il est actuellement admis que l’AP est une pathologie qui touche essentiellement l’adulte jeune [12]. Un article de synthèse des études tunisiennes portant sur l’AP et réalisées entre 2000 et 2009, a conclu que l’âge moyen au moment du diagnostic de la pathologie variait entre 37 et 44 ans [13]. En France, l’âge moyen des travailleurs déclarés pour asthme professionnel, a été de 36 ± 13 ans, avec prédominance de travailleurs âgés de moins de 40 ans (53% des cas) [12]. Dans notre étude, une prédominance féminine (61%) a été notée avec un âge moyen des travailleurs de sexe féminin moins élevé comparativement à ceux de sexe masculin (39 ± 6,7 ans versus 47,7 ± 8,1 ans). De plus, les 3/4 des travailleurs de notre série réparés pour AP (74,3%) exerçaient dans le secteur de textile-confection. Or, il est évident que la nature des industries implantées dans chaque région conditionne en grande partie la répartition des travailleurs selon le sexe. Ainsi la prédominance féminine dans notre série est, en partie, expliquée par l’importance du secteur de confection et textile dans le centre tunisien. Le secteur de textile et de confection est particulièrement bien développé dans la région du centre tunisien. En effet, 25% de la main d’œuvre industrielle du gouvernorat de Monastir est employée dans ce secteur [14].

Dans l’étude de Debbabi et al., le mesurage du niveau d’empoussièrement dans une entreprise de textile installée au centre a conclu à des chiffres élevés dans les unités de filature (1,44 mg/m^3^), de tissage (1,24 mg/m^3^) et de teinture (0,88 mg/m^3^). Ces niveaux dépassent les normes fixées par l’OMS et par l’administration de la sécurité et de la santé au travail (OSHA) [15]. De plus, d’après une étude ergonomique auprès de dix entreprises de textiles dans la région de Monastir, l’hygiène atmosphérique a été défaillante dans toutes les situations du travail dans la branche de filature et dans six situations parmi huit dans les branches de tissage et de finissage [16]. Un terrain atopique a été retrouvé chez 30,2% de nos travailleurs avec une rhinite allergique comme traduction clinique dans 13,2% des cas. Beaudin définit l’atopie comme une facilité anormale de synthétiser les anticorps IgE spécifiques vis-à-vis des allergènes ayant pénétré dans l’organisme par des voies naturelles [17]. Certains auteurs s’accordent que le risque de survenu d’AP est plus élevé parmi les travailleurs atopiques [14]. Parmi les 57 travailleurs chez qui nous avons pu préciser la notion de tabagisme, 12 ont été des fumeurs. Plusieurs études ont montré que le tabagisme augmente le risque de sensibilisation et de développement de l’asthme dans différentes professions [18]. En effet, l’effet irritant du tabac sur la muqueuse bronchique a été suspecté d’augmenter la perméabilité de l´épithélium aux substances inhalées avec facilitation de leur accès aux cellules immunocompétentes [19]. Dans notre étude, une gêne respiratoire isolée a été retrouvée chez 38% des travailleurs. Cette gêne a été associée à une rhinite allergique dans 37,2% des cas, et à une conjonctivite dans 2,3%. L’association entre l’asthme et la rhinite allergique définie par des manifestations cliniques faites de prurit et/ou obstruction nasale, éternuement et rhinorrhée aqueuse est estimée, selon des récentes études, à 80% [20–22]. Une plus grande sévérité des symptômes d’asthme chez les patients ayant une rhinite allergique a été rapportée avec une corrélation positive entre l’intensité des symptômes d’asthme et de la rhinite [23]. La rhinite professionnelle allergique est souvent sous diagnostiquée malgré qu’il s’agisse d’un signe prédictif de l’asthme professionnel [13, 20]. En effet, une histoire de rhino conjonctivite précède souvent le développement de l’AP particulièrement pour les agents de haut poids moléculaire [24]. Dans notre étude, une obstruction nasale a été objectivée par la rhinomanométrie antérieure sans test de provocation associé chez 46% d’entre eux. Selon une étude réalisée dans une entreprise de textile à Monastir, 46% des travailleurs présentaient une symptomatologie de rhinite, associés à un asthme dans 2 cas [15]. Le diagnostic de l’AP est établi grâce à un faisceau d’arguments et de tests concordants qui ne sont pas chacun en soi, une référence exclusive [25].

Dans notre série, le seul examen complémentaire pratiqué chez l’ensemble des patients a été la spirométrie de base. Cette exploration fonctionnelle n’a objectivé de syndrome obstructif réversible que dans 56,6% des cas, alors qu’elle a été normale dans 31% des cas. Quatre déclarations d’AP, faites par le même médecin du travail ont été associées à une débimétrie longitudinale faite par le one flow (débimétrie électronique) objectivant une variabilité du débit expiratoire de pointe (DEP) intra journalière. Le suivi et les mesures itératives du débit expiratoire de pointe ont été proposés par Burge depuis 1979. Récemment Moore et al, à travers une revue systématique de la littérature, ont conclu que des mesures sériées de DEP est faisable, sensitif et spécifique pour le diagnostic d’asthme professionnel [9]. Dans notre série 49 patients ont bénéficié d’un test d’hyper-réactivité bronchique non spécifique (HRNS) à la métacholine, qui se sont révélés positifs dans le total. Des mesures répétées de la réactivité bronchique non spécifique, pendant des périodes d’activité professionnelle et pendant des périodes de non exposition, peuvent aider à établir la relation entre le travail et l’asthme [26]. De plus, il semble que c’est plus aisé de démontrer une augmentation de la réactivité bronchique non spécifique pendant une période de l’exposition à l’agent causal, que d’objectiver sa diminution lors d’une période d’éviction vu que l’amélioration peut ne se manifester que très tardivement [26]. Selon Cartier et al., un test de provocation non spécifique pendant l’activité professionnelle permet d’éliminer le diagnostic d’AP (valeur prédictive négative 90,7%) [27]. La difficulté réside alors dans la hiérarchisation et la rationalisation de la demande de ces explorations. L’adoption d’une démarche diagnostique plus rigoureuse semble être nécessaire, particulièrement face aux conséquences socioprofessionnelles du diagnostic d’une telle pathologie touchant fréquemment des sujets jeunes, et impliquant des pertes d’emploi dans 30 à 40% des cas et des pertes salariales moyennes de 50% du salaire net [28].

### Démarche de réparation

Dans notre étude, l’indemnisation simultanée des séquelles de l’asthme et de rhinite a été accordée à 37% des travailleurs. Les taux d’IPP accordés aux 129 patients indemnisés pour AP au centre tunisien durant les huit ans d’étude était de 26,3% ± 13,8% (10 - 70%) avec une différence statistiquement significative entre les taux accordés par les commissions de Sousse et ceux accordés par la commission de Monastir (p = 0,003). L’étude analytique a conclu à l’absence de corrélation entre les taux d’IPP d’une part et le sexe, le secteur d’activité, le poste occupé, l’ancienneté au poste, l’association ou non de rhinite ou de conjonctivite, les données de l’examen clinique, et les résultats des explorations fonctionnelles notamment ceux de la spirométrie et du test HRNS d’autre part. Seuls l’âge et la commission médicale accordant le taux d’IPP se sont dégagés comme facteurs corrélés à ce taux. Les populations de travailleurs réparés par la commission de Sousse et celle de Monastir, ont été statistiquement comparables, en termes de répartition selon le sexe, les secteurs d’activité, les postes occupés ainsi qu’en terme des données cliniques et résultats d’explorations fonctionnelles et des examens complémentaires. Les systèmes de reconnaissance et d’indemnisation des pathologies professionnelles varient d’un pays à l’autre. Et bien que tous ces organismes médico-légaux reconnaissent l’AP, l’indemnisation offerte demeure largement insuffisante dans la majorité des cas [28]. En Tunisie, parmi les 85 tableaux de la liste officielle des maladies professionnelles, 23 permettent de réparer l’AP. De plus selon le système tunisien, les sujets déclarés bénéficient de « la présomption légale d’origine », reste que la reconnaissance et l’indemnisation de l’AP doit passer par la confirmation du diagnostic de l’asthme par les explorations respiratoires; la confirmation - lors d’enquêtes techniques conduite sur les lieux du travail - de l’exposition à un agent figurant dans l’un des tableaux des maladies professionnelles comme agent étiologique potentiel d’asthme, par le respect des délais de prise en charge, ainsi que par l’inclusion des travaux effectués dont la liste indicative ou limitative sur le tableau en question. En France le système de déclaration et de réparation est comparable au système tunisien avec deux listes officielles de pathologies professionnelles pour les travailleurs agricole et le système général. Néanmoins un recours a une déclaration de cas d’AP « hors liste » reste possible [29].

Concernant la détermination du taux d’IPP, elle est fixée en Tunisie, conformément au barème indicatif des taux d’IPP élaboré et adopté suite à un arrêté des ministres de la santé publique et des affaires sociales. Ce barème tient compte des degrés de l’insuffisance respiratoire évaluée en fonction de la gêne fonctionnelle, des anomalies radiologiques et des anomalies de l´épreuve fonctionnelle respiratoire (la capacité vitale, l´indice de Tiffeneau). Ce barème, comme la majorité des échelles existantes pour la réparation des pathologies respiratoires professionnelles ont été inspirées de celui proposé par l’Association Médicale Américaine. Ces échelles, comme le barème tunisien, semblent plus adaptées à la réparation des pathologies respiratoires responsable de troubles ventilatoires restrictifs, de perturbation de la diffusion gazeuse, ou d’altération des propriétés du parenchyme [28]. C’est pourquoi ces dernières années, beaucoup de critiques ont été portées à ces échelles jugées inadaptées à la réparation des incapacités secondaires à l’asthme professionnel [28]. Au Québec un barème destiné spécifiquement à l’indemnisation de l’AP a été introduit et légalement adopté depuis 1984 [30]. Cette échelle prend en compte le niveau de l’obstruction, le degré de réversibilité, et les traitements nécessaires pour le patient. A coté d’une compensation spécifique des séquelles asthmatiques fixée ultérieurement, un programme d’indemnisation avec remplacement du revenu a été mis en place. En Tunisie, en 2004, l’Institut de Santé Sécurité au Travail (ISST) a proposé un schéma de barème consensuel d’indemnisation de l’AP, fondé sur l’avis d’un collège d’experts tunisiens. Selon ce barème consensuel, le taux d’IPP est fixé en fonction du degré d’obstruction et/ou d’hyper-réactivité bronchique, des besoins en médicaments et d’un coefficient professionnel [31]. Cependant ce barème n’a pas été jusqu´à ce jour officialisé et adopté par un texte légal. Par ailleurs, en plus du barème et des critères d’évaluation adoptés, le moment de fixation du taux d’IPP est déterminant, étant donné le caractère variable de l’obstruction bronchique et du besoin en traitement chez le patient asthmatique. Idéalement ce taux est fixé lorsque l’asthme est jugé raisonnablement contrôlé. Et même si pour certains patients, ce niveau satisfaisant de contrôle de la pathologie serait difficile à obtenir, en général ce contrôle reste possible particulièrement grâce aux efforts conjugués d’équipes pluridisciplinaires [28]. Selon certains auteurs, ce taux sera idéalement fixé deux ans après l’arrêt de l’exposition à l’agent causale vu les améliorations espérées sur le plan clinique et surtout spirométrique. En effet les données de l’exploration fonctionnelle atteignent un plateau après cette durée de retrait de l’environnement causal [28].

## Conclusion

La Rhinite et l’asthme sont rapportés avec un nombre croissant de substances utilisé en milieu professionnel [1]. On estime que 10 à 25% des cas d’asthme de l’adulte peuvent être attribuables à l’activité professionnelle [2]. Cette pathologie, reste souvent sous-estimée et sous- déclarée [14]. Cette étude menée sur huit ans de façon exhaustive sur les cas d´AP indemnisés dans la région du centre Tunisien, a permis d´apprécier les caractéristiques épidémiologiques et cliniques des patients atteints, ainsi que d’évaluer les pratiques des comités habilités à fixer les taux d’IPP et d’objectiver les discordances d’application du barème indicatif officiel.

### Etat des connaissances actuelles sur le sujet

- L’asthme professionnel compte parmi les pathologies respiratoires les plus fréquentes en milieu de travail;
- Il s'agit d'une maladie qui affecte le sujet jeune actif caractérisée par de lourdes conséquences médicales et socio-économiques, avec un pronostic médical qui peut être réservé même après suppression de l’exposition à l’agent causal une perte d’emploi et une perte salariale souvent rapportées.

### Contribution de notre étude à la connaissance

- Cette étude a permis d'apprécier les caractéristiques épidémiologiques et cliniques des patients atteints;
- Elle a également pu évaluer les pratiques des comités habilités à fixer les taux d'incapacité partielle permanente et d’objectiver les discordances d’application du barème indicatif officiel incitant à la création d'un barème de consensus.
